# Induced oscillatory signaling in the beta frequency of top-down pain modulation

**DOI:** 10.1097/PR9.0000000000000806

**Published:** 2020-01-17

**Authors:** Martin Diers, Cecile C. de Vos, Wiebke Gandhi, Marie E. Hoeppli, Susanne Becker, Elisabeth Bock, Sylvain Baillet, Petra Schweinhardt

**Affiliations:** a Department of Psychosomatic Medicine and Psychotherapy, LWL University Hospital, Ruhr University Bochum, Bochum, Germany; b Faculties of Medicine and Dentistry, McGil University, Montreal, QC, Canada; c Centre for Pain Medicine, Erasmus Medical Centre, Rotterdam, the Netherlands; d McConnell Brain Imaging Centre, Montreal Neurological Institute, McGill University, Montreal, QC, Canada; e School of Psychology and Clinical Language Sciences, Centre for Integrative Neuroscience & Neurodynamics (CINN), University of Reading, Reading, United Kingdom; f Department of Anesthesiology, Cincinnati Children's Hospital Medical Center, Cincinnati, OH, USA; g Department of Cognitive and Clinical Neuroscience, Medical Faculty Mannheim, Central Institute of Mental HealthHeidelberg University, Mannheim, Germany; h Department of Chiropractic Medicine, Integrative Spinal Research Group, University Hospital Balgrist, University of Zurich, Zurich, Switzerland

**Keywords:** Attention, Pain modulation, Magnetoencephalography, Electrical intraepidermal stimulation

## Abstract

**Background:**

Induced synchronized brain activity, particularly in the beta-frequency range, has rarely been investigated in human electrophysiological studies of attentional modulation of the perception of nociceptive stimuli.

**Methods:**

We measured time-resolved brain responses to nociceptive stimuli in healthy subjects (final data set: n = 17) using magnetoencephalography (MEG). In addition to investigating evoked responses as previous studies, we tested whether synchronized beta activity induced by nociceptive stimuli differs between 2 attentional conditions. Subjects were presented simultaneously with 2 stimulus modalities (pain-producing intraepidermal electrical stimuli and visual stimuli) in 2 different experimental conditions, ie, “attention to pain” and “attention to color.” Pain ratings between conditions were compared using a 2-sided paired-sample *t* test; MEG data were analyzed with Brainstorm.

**Results:**

Pain ratings were significantly higher in the “attention to pain” compared with the “attention to color” condition. Peak amplitudes of the evoked responses were significantly larger in the “attention to pain” condition bilaterally in the insula and secondary somatosensory cortex, and in the primary somatosensory cortex (SI) contralateral to stimulation. Induced responses to painful stimuli were significantly stronger in contralateral SI in the beta-frequency range in the “attention to pain” condition.

**Conclusions:**

This study replicates previous reports w.r.t. the attentional modulation of evoked responses and suggests a functional role of induced oscillatory activity in the beta frequency in top-down modulation of nociceptive stimuli.

## Abstract 2

Supplemental Digital Content is Available in the Text.

## 1. Introduction

Attentional modulation of nociceptive stimuli is an extensively researched form of cognitive-emotional pain modulation. In the framework of the limited-capacity model of human cognition, positing that concomitant sensory inputs compete for brain processing capacity,^6^ the relative importance ascribed to sensory inputs is influenced by both bottom-up stimulus saliency and top-down processes.^11^ In line with top-down processes influencing the relative importance, it has repeatedly been shown that nociceptive stimuli are perceived as more painful and associated with enhanced activation in pain-related brain regions when attention is directed towards them [reviewed in Refs 27 and 44]. This is also observed in intermodal experimental paradigms when the attentional focus is changed between sensory modalities while bottom-up characteristics of sensory inputs are kept constant across experimental conditions.^33,43^ An early electrophysiological study in nonhuman primates^9^ showed increased second-order neuron activity of afferent pathways when the animals attended to the nociceptive stimuli vs when they attended visual stimuli, indicating top-down effects of shifting the attentional focus.

In addition to informing about activation magnitude, which is also achieved using brain imaging modalities, scalp electroencephalography (EEG) or magnetoencephalography (MEG) have the potential to discriminate between multiple signaling mechanisms that mediate sensory processing because of their high temporal resolution.^1^ Recent data from the visual system have suggested a possible role for beta-range activity in the top-down modulation of stimulus perception.^3,34,40^ Conversely, gamma-range activity is involved in the selection of salient stimuli^17^ and may convey bottom-up signaling in large-scale brain networks.^3^

Here, we sought to interrogate oscillatory beta signaling as a marker of top-down modulation of the processing of nociceptive stimuli. In addition, we compared the evoked responses in pain processing and top-down attention modulating areas between attention and distraction conditions for comparison with previous studies. We used MEG source imaging with an adapted intermodal paradigm previously used psychophysically^33,46^ and with functional magnetic resonance imaging (fMRI).^45^

## 2. Material and methods

### 2.1. Subjects

Previous studies using the experimental design used here reported medium to large behavioral effects of attentional focus on/away from nociceptive stimuli in samples of 7 to 15 subjects.^33,45,46^ We therefore recruited 20 healthy subjects for this study. Exclusion criteria were any present or past pain condition, psychiatric disorders, substance abuse behaviors, regular night shifts, sleep disorders, claustrophobia, pregnancy, or red-green color weakness tested with the Ishihara test for color perception.^24^ All subjects had normal or corrected-to-normal vision. The study was approved by the McGill University Institutional Review Board, and informed consent was obtained from all subjects according to the revised Declaration of Helsinki.

### 2.2. General design

Subjects attended one experimental MEG session, followed by the acquisition of an anatomical MRI for coregistration with the MEG data. The experiment followed a within-subject repeated-measures design with “condition” (2 levels: “attention to pain” and “attention to color”) as within-subject factor. To exclude any activation difference between the conditions due to different sensory inputs, identical stimuli (pain-inducing intraepidermal electrical stimuli and colored circles) were presented in the 2 conditions. The only difference between conditions was the attentional focus: in the “attention to color” condition, subjects were instructed to detect a deviation from the standard transparency of the colored circle (see Experimental task); in the “attention to pain” condition, they were instructed to detect a deviation from the standard intensity of the electrical stimulation.

### 2.3. Experimental task

**Figure 1.**
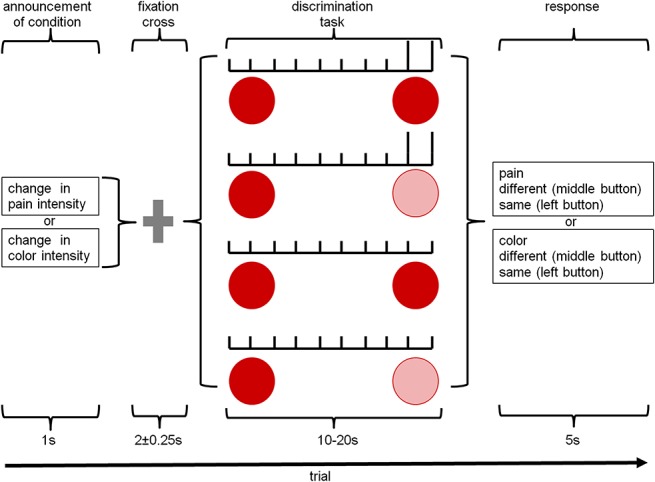
Study design: after indicating the condition of the trial and a fixation cross, the standard circle (solid red) was projected and, simultaneously, 3 to 8 standard electrical stimuli (short vertical bars) were applied, followed by 2 standard or deviant (longer vertical bars) electrical stimuli and the standard or deviant circle (light pink)—ie, four different combinations were possible. The trial finished with a response of the subject to the discrimination task.

Each trial (Fig. 1 and Table 1) started with a slide instructing the subject to pay attention to changes in color intensity (“attention to color”) or in electrical stimulus intensity (“attention to pain”). Before the start of the experiment, subjects were told, that the intensity of both could change at any time. These instructions were presented for 1000 ms followed by a fixation cross for 2000 ± 250 ms. Then, a circle of standard transparency (see 2.5) was displayed, and between 3 and 8 electrical stimuli of standard intensity were delivered. Then, 2 additional standard or deviant electrical stimuli were delivered, and the transparency of the circle changed or remained unchanged. Hence for each condition, 4 different trial endings were possible: (1) deviant electrical stimulation with standard color, (2) deviant electrical stimulation with deviant color, (3) standard electrical stimulation with standard color, and (4) standard electrical stimulation with deviant color. The reason for varying the number of electrical stimuli and the length of the display of the circle of standard transparency was to keep the time point of a potential deviant stimulus unpredictable. Every condition (“attention to color” and “attention to pain”) had the same number of deviant electrical and visual stimuli. Four trials of the same condition were presented in a block, and each of the 4 trial endings occurred once in each block in a pseudorandomized fashion. The trial ended with a response to the discrimination task (“same or different color intensities” or “same or different pain intensities”). The discrimination task was used to ensure that subjects had shifted their attention to the instructed condition.^33^ A block lasted approximately 120 seconds and at the end of each block, subjects rated the average pain caused by the electrical stimulation during the block using a LUMItouch button box (Photon Control, Inc, Burnaby, BC, Canada). Six blocks of each condition were presented in pseudorandom order (12 blocks in total). The total time of the experimental task was approximately 24 minutes.

**Table 1:**
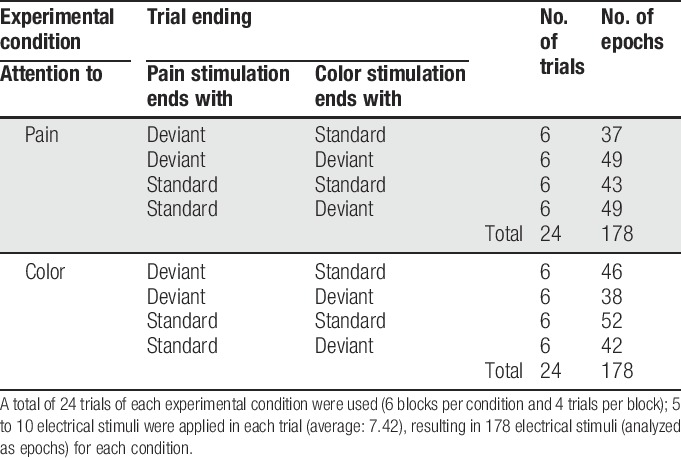
Overview of the conditions and the number of trials and epochs per condition.

### 2.4. Painful electrical stimulation

Intraepidermal electrical stimulation of the thumb, preferably activating Aδ fibers^22,23,48^ was used to induce pain. Because attentional processing is expected to present with a right-hemispheric dominance,^11^ stimulus delivery was to the right thumb to allow for a better distinction between pain processing in contralateral (ie, left) primary somatosensory cortex and attentional processing in the right hemisphere. Disposable stainless steel needle electrodes were used with a 0.35-mm uninsulated tip, 2-mm^2^ stimulation area, and 0.5-cm separation between the electrodes (model: 9013R0272, 28G; Alpine Biomed ApS, Skovlunde, Denmark). An electrical stimulus consisted of a double pulse of 1.5-ms duration in total with a 1-ms interpulse interval. Electrical stimuli were presented at 2000 ± 250-ms interstimulus intervals. A constant current stimulator (BSL MP30—BSLSTMA, Biopac Systems, Inc, Aero Camino Goleta) was located outside the MEG room, and the electrodes were connected to the stimulator through an extension cable. Delivery of the electrical stimuli was controlled by Presentation (Neurobehavioral Systems, Inc, Berkeley, CA) and a NI USB-6259 M series high-speed multifunction data acquisition module (National Instruments, Austin, TX).

Individual perception thresholds and pain thresholds were determined before MEG data collection by averaging the last 4 thresholds of 6 alternating ascending and descending series. Throughout the ascending series, subjects indicated when they first felt something (perception threshold) and when they first perceived the sensation as painful (pain threshold) (and vice versa for descending series). Pain tolerance was assessed with 3 ascending series, averaging the stimulus intensities judged as “highest intensity tolerable” of the last 2 series. To determine the intensity of the standard electrical stimuli, the intensity was set at 50% between the intensity of the individual's pain threshold and pain tolerance and adjusted until the subject rated it as 150 to 160 on the intensity scale (moderate pain, see 2.6). To determine the intensity of the deviant electrical stimuli, the intensity was set 30% higher than the standard stimulus intensity and adjusted in test trials to identify the smallest increment in intensity that was still noticeable by the subject.

### 2.5. Visual stimuli

Visual stimuli were displayed on a video back-projection screen (50-cm distance to the subject). An opaque red circle (red/green/blue: 255/0/0) was used. The deviant circle had the same color values but was presented with higher transparency; transparency values with 6 of 10 stimuli correctly detected as different by the subject in test trials were used for the experiment. The administration of visual stimuli was controlled by Presentation (Neurobehavioral Systems, Inc).

### 2.6. Pain ratings

Subjects rated the average perceived pain intensity caused by the electrical stimulation during a block using a visual analogue scale (with the anchors 0 “no sensation,” 100 “pain threshold”, ie, the slightest perception of pain, and 200 “most intense pain tolerable”).^4,5,29,46^

### 2.7. Magnetoencephalography and magnetic resonance imaging

**Figure 2.**
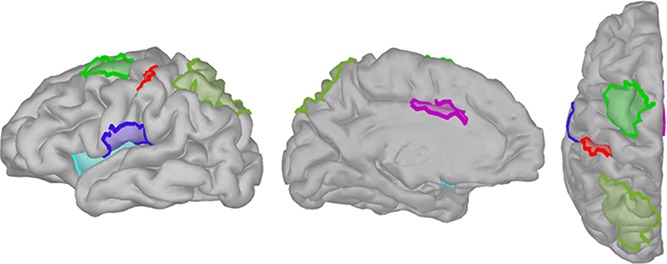
Regions of interest (ROIs) were defined on the individual brains, provided is an example of one individual. Middle-anterior cingulate cortex (aMCC) (pink), primary somatosensory cortex (SI) (red), secondary somatosensory cortex (SII) (dark blue), insular cortex (light blue), superior parietal cortex (SPC) (light green), and frontal eye fields (FEF) (dark green).

MEG recordings were obtained from 275 axial gradiometers (CTF; Coquitlam, BC, Canada), with third-order gradient compensation and a sampling rate of 2400 Hz with a 600-Hz antialiasing filter. Electro-oculogram (EOG) and electrocardiogram (ECG) were recorded to capture eye blinks and cardiac activity, following guidelines of good MEG practice.^19^ Additional head-positioning coils were taped to the scalp to monitor head movements. Using a 3-D digitizer system (Fastrack; Polhemus, Colchester, VT), the coil locations and 100 scalp points were collected for coregistration of the MEG data with the subject's anatomical MRI scan (high white-gray matter contrast T1-weighted MRI scan; 3D gradient echo sequence, TR = 2420 ms, TE = 3.7 ms, flip angel = 9°, inversion time = 960 ms, 240 × 240-mm field of view, one hundred twenty four 1.3-mm axial slices; 1.5T Siemens Sonata, Siemens Medical Systems, Erlangen, Germany). Individual cortical and scalp surfaces were obtained using FreeSurfer (https://surfer.nmr.mgh.harvard.edu/). All MEG data analysis and registration with MRI were performed with Brainstorm.^41^ The cortical surfaces were down-sampled to about 15,000 vertices to constrain distributed MEG source models. Individual cortical surfaces were used to define 7 regions of interest (ROIs) involved in pain processing^16^: middle-anterior cingulate cortex (aMCC), primary somatosensory cortex (SI) (bilateral), secondary somatosensory cortex (SII) (bilateral), and insular cortex (bilateral). Region of interest definition was achieved by importing Destrieux atlas provided in FreeSurfer into Brainstorm (Fig. 2). For SI, we selected only the area of the hand knob. In addition, 4 bilateral ROIs were defined as areas implicated in top-down attentional processes^11,28,43^: superior parietal cortex and frontal eye fields (FEF). Because definition of the FEF using macroanatomical landmarks is difficult,^44^ it was defined using the Neurosynth database.^49^ For this, cortical surfaces around the most significant MNI coordinates for the term “frontal eye” were grown until the FEFs contained about 170 cortical vertices for every subject, corresponding to approximately 24 cm^2^. For the SPCs, the superior parietal cortex from the Destrieux atlas provided in FreeSurfer^14^ was imported in Brainstorm.

### 2.8. Data analysis

#### 2.8.1. Behavior

The ratings of the electrical stimuli were averaged across the 6 blocks of each condition for each subject and compared between conditions using a two-sided paired-sample *t* test.

The answers of the discrimination task after each trial (change in intensity of the attended stimulus) were recorded, and the percentages of correctly detected changes in pain or color intensity were calculated for each subject.

#### 2.8.2. Magnetoencephalography

Power-line external noise on the MEG signals was removed using notch filters around 60, 120, and 180 Hz. After visual inspections of raw recordings, eye blinks, saccades, and cardiac events were detected automatically with Brainstorm, and the related signal artifacts were attenuated using one-dimensional signal-space projectors for each type of artefact.^36^ Data were epoched over a time window of −200 to 1500 ms relative to each electrical stimulus at 0 ms. For each condition, MEG data from 178 epochs were extracted. An average of 5 (SD: 6) epochs for the pain condition and 6 (SD: 8) epochs for the color condition were discarded per subject due to visible movement artefacts. Source models of sensor data were derived with Brainstorm, using the overlapping-spheres approach for head modelling, and distributed source modeling with Brainstorm's weighted minimum-norm estimator constrained to the individual subject's cortical surface, both with default parameters. The amplitudes of the source time series were *z*-scored using the 200 ms before the stimulus as baseline, to standardize responses across ROIs and subjects. To study differences in evoked responses between the 2 conditions (attention to color and attention to pain), the individual evoked responses to the electrical stimuli (standard and deviant) were averaged for each pain-related ROI and for each condition across all subjects. The peak amplitudes of evoked responses were compared between conditions using a two-way repeated-measures analysis of variance with “condition” (2 levels) and “ROI” (7 levels) as within-subject factors. Because sphericity could not be assumed (Mauchly's test *P* > 0.05) and Greenhouse–Geisser Epsilon was greater than 0.75, Huynh–Feldt results are reported. Post hoc comparisons were performed using paired *t*-tests. Statistical analyses of the evoked responses were performed using SPSS Statistics Version 23 (IBM), and the threshold for statistical inference was set at α = 0.05.

To identify possible induced power fluctuations of cortical responses across frequencies, we obtained time–frequency decompositions of all time series (−200 to 1500 ms) in each ROI and for every individual trial (range: 1–150 Hz, Morlet wavelets with central frequency 1 Hz and full-width half-maximum of 3 seconds, with wavelet coefficients *z*-score standardized with respect to prestimulus baseline [−200 to −5 ms]). The individual TFMs were averaged for each ROI and for each condition across all subjects. Frequency bands were interpreted as follows: 1 to 4 Hz—delta, 4 to 8 Hz—theta, 8 to 12 Hz—alpha, 15 to 35 Hz—beta, and 40 to 80 Hz—gamma. The time–frequency maps of all ROIs were compared between the 2 conditions using Brainstorm's FDR-corrected permutation tests (α = 0.05^37^).

## 3. Results

One subject withdrew from the study because of fear of the intraepidermal needle; 2 subjects were excluded from analysis because of movement and stimulation artefacts that could not be corrected without compromising the MEG data. Therefore, data from 17 subjects are presented (9 women, average age [SD]: 24 [4] years).

### 3.1. Behavioral data

#### 3.1.1. Electrical and visual stimulation

The average intensity of the standard electrical stimuli was 2.2 mA (SD: 0.7 mA, range: 1.3–3.9 mA), corresponding to, on average, 5 times the subjects' sensation threshold. The average intensity of the deviant electrical stimuli was 2.9 mA (SD: 1.0 mA, range: 1.7–5.6 mA). The opacity of the standard visual stimulus was 100% and the deviant opacity of the circle was on average 75% (SD: 2%, range: 71%–78%).

#### 3.1.2. Task performance

For one subject no responses to the discrimination task were recorded due to computer problems and one subject reported partly pressing the wrong buttons. Although not included in the analysis of the discrimination task, their MEG and pain rating data were nevertheless used because the exit interviews indicated that they performed the experiment according to protocol. The remaining 15 subjects detected changes in the pain or color stimuli correctly in at least 75% of the trials, with on average 79% (SD = 14%) correct in the “attention to pain” condition and 89% (SD = 8%) correct in the “attention to color” condition. Although these rates differ (*P* = 0.03), they indicate that subjects were paying attention to the respective condition as intended by the experimental paradigm. The individual accuracy rates were stable throughout the experiment (slope of linear trend analysis: *x* = 0.01).

#### 3.1.3. Pain ratings

The average pain rating of the 17 subjects for the “attention to pain” condition was 136 (SD: 15) and for the “attention to color” condition 132 (SD: 18) (t = −2.25, *P* = 0.039, Cohen's d = 0.3, achieved power = 0.323).

### 3.2. Evoked responses in pain processing areas

**Figure 3.**
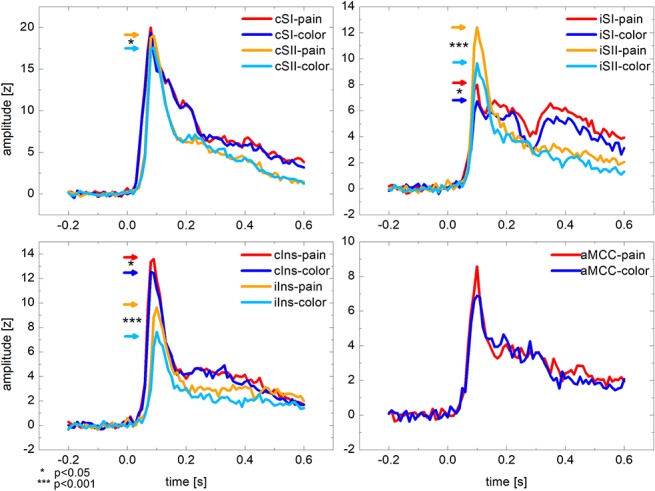
Evoked responses (*z*-scores) in pain processing ROIs (SI, primary somatosensory cortex; SII, secondary somatosensory cortex; Ins, insula; aMCC, middle-anterior cingulate cortex; c, contralateral to the stimulation; i: ipsilateral to the stimulation), averaged across all subjects for the “attention to pain” (“pain”) and the “attention to color” (“color”) condition. Displayed epochs are −200 to 600 ms relative to the electrical stimuli.

For every subject and every ROI, the amplitude of the evoked response peaked during the first 50 to 150 ms after stimulation. The largest responses to the electrical stimuli were in SI and SII contralateral to the stimulation (ie, in the left hemisphere). For all ROIs, the peak amplitudes in the “attention to pain” condition were higher compared with the “attention to color” condition (Fig. 3). The repeated-measures analysis of variance on the peak amplitudes showed a significant main effect of condition (F(1,16) = 30.2, *P* < 0.0001), and post hoc *t*-tests indicated significant effects for contralateral and ipsilateral insula, contralateral and ipsilateral SII, ipsilateral SI, and a trend for ACC (Table 2).

**Table 2:**
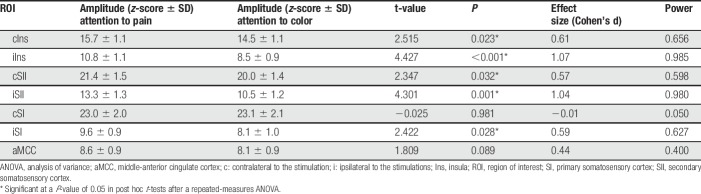
Effect of experimental condition on the peak amplitudes of the evoked responses.

### 3.3. Time–frequency maps

**Figure 4.**
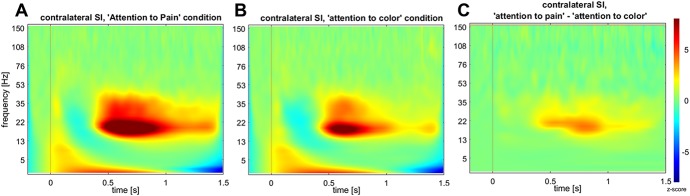
Average time–frequency map (frequency range 1–150 Hz) from −200 to 1500 ms of contralateral (left) SI for (A) “attention to pain” condition, (B) “attention to color” color condition, and (C) “attention to pain” minus “attention to color” (*z*-scores).

**Figure 5.**
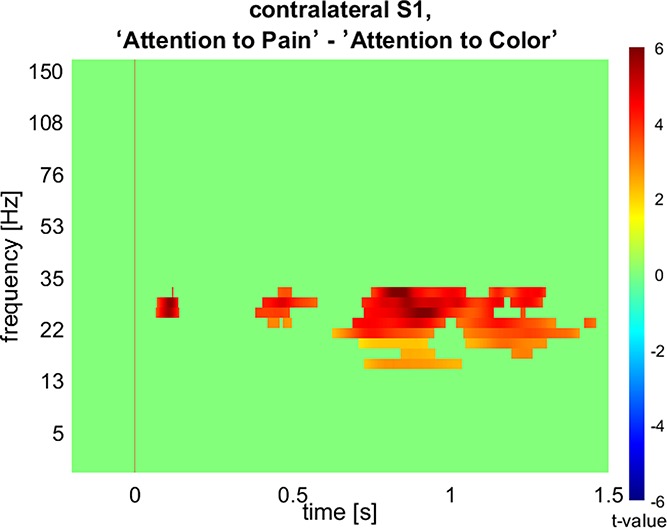
The result of the permutation *t* test (FDR corrected for multiple ROIs) on the contralateral SI time–frequency map for the difference between the “attention to pain” condition and the “attention to color” condition indicates statistically significant differences between the 2 conditions in the beta band at several time points; red represents increased activity in the “attention to pain” condition; and blue indicates increased activity in the “attention to” condition.

During the first 150 ms after application of the painful electrical stimulus, activity increased in a broad frequency range, from 1 to 100 Hz, in all pain-related and attention-related ROIs. The largest activity in both conditions was found in contralateral SI between 12.5 and 40 Hz (Fig. 4A, B), which corresponds predominantly to beta-band activity. There was an initial beta-activity increase (0–150 ms), followed by beta suppression (150–400 ms) and a strong beta rebound (400–1000 ms). The largest difference between the 2 conditions was found also for the beta band in contralateral SI (Fig. 4C). The difference in response between the 2 conditions appeared particularly strong in the beta-rebound phase. This was confirmed by FDR-corrected permutation tests (*P* < 0.05) on the time–frequency maps of the 7 pain-related ROIs, which showed significantly stronger beta-range activity in the pain condition in contralateral SI after 100 to 200 ms (*t* = 5.9), 350 to 600 ms (*t* = 4.8), and 700 to 1000 ms (*t* = 6.3) (Fig. 5). The time–frequency maps of any other region did not significantly differ between the 2 conditions.

In both conditions (attention to color and attention to pain) induced gamma-band activity (50–90 Hz, with 60 Hz being notch filtered) was observed between 50 and 250 ms in contralateral SI, albeit below statistical significance (supplementary figure, available at http://links.lww.com/PR9/A61). There were no significant differences in the gamma band between the 2 conditions.

## 4. Discussion

The largest evoked responses to the painful electrical stimulation, irrespective of condition, were found in SI and SII contralateral to the stimulation. The peak amplitudes of the evoked responses differed significantly between the 2 conditions in most regions tested. Time–frequency analyses showed the strongest induced oscillatory response in contralateral SI in the beta-frequency range, which was significantly stronger in the “attention to pain” condition compared with the “attention to color” condition.

### 4.1. Effects of attention on evoked responses in pain processing regions

In line with previous EEG^12,18,25,26,31^ and MEG studies,^30,32,47^ significant differences of the peak amplitudes between conditions were observed in this study bilaterally in the insula and SII and ipsilaterally in SI. These results are mirrored by fMRI and PET activation studies, describing consistently attentional effects on nociceptive processing in the insula, somatosensory cortices, and aMCC with a range of experimental paradigms.^2,7,8,15,38,39,40^ The observation of attentional modulation of brain activity/activation in a network of cortical pain processing regions is compatible with the view that attentional modulation of nociceptive input is achieved through descending pathways.^43^

### 4.2. Effects of attention on induced beta and gamma activity

We found strong induced activity in the beta range in response to the painful stimulation, which included a strong rebound after beta suppression. This is in line with findings from a previous MEG study from Hauck et al.,^21^ who, however, did not observe an effect of attention on induced beta activity. Here, we report that induced beta activity, and in particular the beta rebound in contralateral SI, was significantly stronger when subjects attended to pain. Hauck et al. did not perform a source localization analysis of the beta activity, affording the possibility that a potential effect of condition in a particular brain region, such as SI, was missed. Alternatively, the different experimental paradigm in which attentional load was altered in addition to attentional focus^21^ might explain the apparent discrepancy with this study. This possibility is supported by an EEG study by Chien et al.,^10^ where the attentional focus was manipulated, similar to this study. They found a significant interaction between task (attention vs distraction) and stimulus modality (painful vs nonpainful) for beta activity.^10^ Yet, the pairwise post hoc comparisons were reported nonsignificant after correction for multiple comparisons. Nevertheless, attended painful stimuli were associated with greater beta activity compared with the nonattended painful stimuli.^10^ This observation, in conjunction with the results of this study, indicates that induced beta activity might indeed be a relevant signal of interest when studying pain modulatory attention effects.

We also observed low levels of induced activity in the gamma band in contralateral SI in response to nociceptive stimuli between 50 and 250 ms after stimulus. Our observations are in line with previous MEG studies^20,21^ but were not statistically significant. Amplitudes of pain-induced gamma oscillations in SI and prefrontal cortex have been shown to vary with subjectively perceived pain intensity,^20,35,50^ in line with the notion that gamma-band activity represents one mechanism of preferred processing of sensory information.^13^ However, if induced gamma activity is related to perceived pain intensity, should it not track perceptual pain modulation by attention? Hauck et al. indeed found stronger induced gamma activity 50 to 250 ms after stimulus when attention was directed towards nociceptive stimuli.^21^ As indicated above, the experimental paradigm of the Hauck study differed in not attempting to keep attentional load constant. In the study by Chien et al.,^10^ in which attentional load was kept constant, induced gamma activity did not differ between attended and unattended painful stimuli (a significant task by stimulus modality interaction was driven by an effect of stimulus modality in the unattended conditions). Similarly, we did not find an effect of experimental condition on induced gamma activity in response to nociceptive stimuli (although this has to be interpreted cautiously because, in our study, induced gamma activity was below statistical significance in both experimental conditions). Thus, albeit there is evidence that induced gamma-activity tracks perceived pain intensity, it might depend on how variations in perceived intensity are achieved. This idea is supported by an EEG study comparing changes in perceived pain intensity achieved either by varying stimulus intensity (bottom-up modulation) or by placebo analgesia (top-down modulation),^42^ which found that induced gamma activity was influenced by bottom-up modulation but not by top-down modulation.

These findings from the present and other studies indicating different roles for induced gamma and beta activity in the processing of attended and unattended nociceptive stimuli are particularly interesting in light of previously reported data from the visual and auditory systems, where gamma activity was found to be related to bottom-up signaling, and beta-band activity was associated with top-down modulations.^3^ Pain modulation by bottom-up vs top-down processes might be similarly encoded.

This study has several limitations. We used an interstimulus interval of 2 seconds (±250 ms) to be able to analyze epochs up to 1500 ms for the time–frequency maps. At these stimulation rates, habituation effects might occur and impact the attention effects on the stimulus processing. However, there are 2 important considerations: (1) The ISI varied around a mean of 2 seconds with SD of 250 ms with the aim to reduce habituation effects. (2) The ISIs were the same in the 2 conditions, and it is the difference between conditions that is the comparison of interest. Thus, any difference in conditions would have occurred despite a habituation effect. Another point to consider is that the relatively high-stimulation intensities may have coactivated Aß fibers. This might have influenced the SI source. In addition, the small sample size might bias the results. Future studies should investigate this effect in larger samples.

### 4.3. Conclusions

We replicate a series of previous studies by the finding that the peak amplitudes of evoked responses are lower in pain processing regions when nociceptive stimuli are not attended compared with when they are attended. Furthermore, we provide evidence that induced activity in the beta-frequency range in SI is related to attentional modulation of nociceptive stimuli. This fits well to previous literature on vision pointing at differential roles of beta and gamma activity for top-down and bottom-up modulatory processes of different sensory modalities. Thus, this relatively small study might serve as a basis for future in-depth examination of top-down and bottom-up pain modulation and their relationship to beta and gamma activity.

## Disclosures

The authors have no conflicts of interest to declare.

This research was supported by a price from an internal MEG competition, the Deutsche Forschungsgemeinschaft DI 1553/3 to M. Diers, a Merit Scholarship Program for Foreign Students (Ministère de l'Education et de l'Enseignement Supérieur, MELS, Quebec), a Quebec Bio-Imaging Network (QBIN) scholarship for foreign students, a The Louise and Alan Edwards Foundation's Edwards PhD Studentships in Pain Research to W. Gandhi, a Discovery Grant from the Natural Science and Engineering Research Council of Canada (436355-13), a NIH (1R01EB026299-01), and a Platform Support Grant from the Brain Canada Foundation (PSG15-3755) to S. Becker.

## Appendix A. Supplemental digital content

Supplemental digital content associated with this article can be found online at http://links.lww.com/PR9/A61.

## Supplementary Material

SUPPLEMENTARY MATERIAL
