# Supplementary material for: Induced oscillatory signaling in the beta frequency of top-down pain modulation
Source: Pain Rep. 2020 Jan 17;5(1):e806. doi: 10.1097/PR9.0000000000000806 (PMC7004500; doi:10.1097/PR9.0000000000000806)

Supplementary figure

*Average time-frequency map (frequency range 50 to 150Hz) from -200 to 1500 ms of contralateral (left) SI for A. ‘attention to pain’ condition, B. ‘attention to color’ condition, C. ‘attention to pain’ minus ‘attention to color’ (z-scores).*


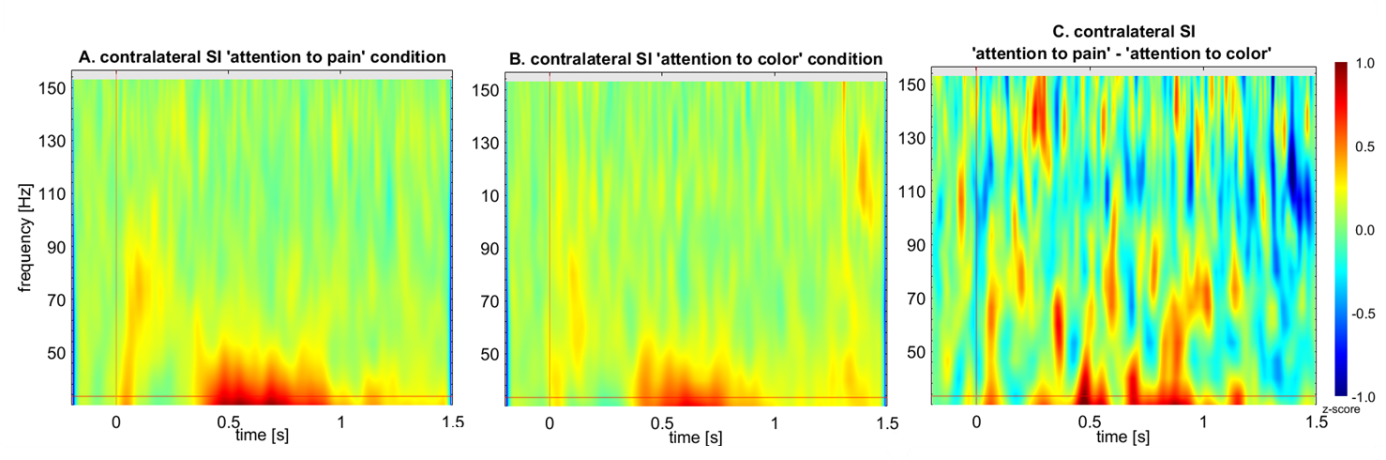

Supplement: SUPPLEMENTARY MATERIAL [file painreports-5-e806-s001.docx]
